# Levosimendan in patients with left ventricular dysfunction undergoing cardiac surgery: a meta-analysis and trial sequential analysis of randomized trials

**DOI:** 10.1038/s41598-018-26206-w

**Published:** 2018-05-17

**Authors:** Zhenhua Xing, Liang Tang, Pengfei Chen, Jiabing Huang, Xiaofan peng, Xinqun Hu

**Affiliations:** Department of Cardiovascular Medicine, The Second Xiangya Hospital, Central South University, Changsha, Hunan 410011 China

## Abstract

Patients with left ventricular dysfunction (LVD) undergoing cardiac surgery have a high mortality rate. Levosimendan, a calcium sensitizer, improves myocardial contractility without increasing myocardial oxygen demand. It is not clear whether levosimendan can reduce mortality in cardiac surgery patients with LVD. The PubMed, Embase, and Cochrane Central databases were searched to identify randomized trials comparing levosimendan with conventional treatment in cardiac surgery patients with LVD. We derived pooled risk ratios (RRs) with random effects models. The primary endpoint was perioperative mortality. Secondary endpoints were renal replacement treatment, atrial fibrillation, myocardial infarction, ventricular arrhythmia, and hypotension. Fifteen studies enrolling 2606 patients were included. Levosimendan reduced the incidence of perioperative mortality (RR: 0.64, 95%CI: 0.45–0.91) and renal replacement treatment (RR:0.71, 95%CI:0.52–0.95). However, sensitivity analysis, subgroup analysis and Trial Sequential Analysis (TSA) indicated that more evidence was needed. Furthermore, levosimendan did not reduce the incidence of atrial fibrillation (RR:0.82, 95%CI:0.64–1.07), myocardial infarction (RR:0.56, 95%CI:0.26–1.23), or ventricular arrhythmia (RR:0.74, 95%CI:0.49–1.11), but it increased the incidence of hypotension (RR:1.11,95%CI:1.00–1.23). There was not enough high-quality evidence to either support or contraindicate the use of levosimendan in cardiac surgery patients with LVD.

## Introduction

More than 1 million patients undergo cardiac surgery with cardiopulmonary bypass (CPB) in the United States and Europe every year^1^. Despite the reduction in perioperative mortality observed over the past two decades, the risk of cardiac surgery in patients with severe left ventricular dysfunction (LVD) remains high, especially with regard to the likelihood of developing postoperative low cardiac output syndrome (LCOS)^2^. Up to 20% of patients developed acute perioperative LVD after cardiac surgery^3,4^, which is a major complication of cardiac surgery and increases mortality significantly^5^. This syndrome may necessitate inotropic drugs (catecholamines and phosphodiesterase type 3 [PDE-3] inhibitors) and mechanical circulatory support (intra-aortic balloon pump). Although these agents may increase cardiac output, they are associated with an increase in morbidity and mortality^6,7^. New drugs without obvious adverse effects are needed.

Levosimendan, a calcium sensitizer, improves myocardial contractility without increasing myocardial oxygen demand^8^. It has been proven to be effective by small clinical trials and meta-analyses in patients with LVD undergoing cardiac surgery^9–14^. However, 3 recent, large, randomized, clinical trials (RCT) produced results inconsistent with previous findings and did not found that levosimendan used either prophylactically or after cardiac surgery is effective in reducing mortality in cardiac surgery patients^15–17^. Given the conflicting evidence about the use of levosimendan in patients with LVD undergoing cardiac surgery, we performed this meta-analysis to evaluate the use of levosimendan in patients with LVD undergoing cardiac surgery.

## Methods

### Search strategy and selection criteria

This meta-analysis was performed in accordance of the Preferred Reporting Items for System Reviews and Meta-analyses (PRISMA) Statement^18^. We systemically searched PubMed, Embase, and Cochrane Library for relevant articles published before September 1, 2017. The search term was “levosimendan.” The search was combined with filters to identify RCTs in the PubMed and EMBASE databases. Results were limited to trials published in English. We manually searched the reference lists of relevant studies, reviews, editorials, and letters to identify further articles. We used Endnote (Thompson ISI ResearchSoft, Philadelphia, PA, US) to manage relevant articles and remove duplicate articles.

### Study criteria, quality assessment, and data extraction

Studies were included if they met the following criteria: (1) the study design was a RCT; (2) all patients were with LVD before or after cardiac surgery; (3) patients were randomly assigned to levosimendan group or the traditional treatment group; (4) relevant data were retrievable. The definition of LVD was defined by each included study. When relevant data were missing, authors were contacted by e-mail, before excluding the references for inaccessibility of data.

The primary endpoint was perioperative mortality. Secondary endpoints included renal-replacement therapy, atrial fibrillation, myocardial infarction, ventricular arrhythmia, and hypotension. All clinical endpoints were evaluated according to per protocol definitions. We judged study quality by evaluating trial procedures for random sequence generation (selection bias), allocation concealment (selection bias), blinding of participants and personnel (performance bias), blinding of outcome assessment (detection bias), and incomplete outcome data (attrition bias). The Cochrane Reviewer’s Handbook 4.2 was used to assess risk of bias.

Relevant data abstraction was completed by 2 independent investigators (PF Chen and JB Huang). Disagreements were resolved by consensus or a third investigator (XQ Hu). We abstracted the following data from the selected articles: first author, publication date, study design, characteristics of included participants, total number of levosimendan group and conventional treatment group, events in the levosimendan group and conventional treatment group, duration of follow-up, primary study endpoints, and other key outcomes.

### Data analysis

Reported event frequencies were used to calculate risk ratios (RRs) with 95% confidence intervals (CI). Heterogeneity between studies was checked and quantified using the I^2^ statistic, with I^2^ < 25% considered low and I^2^ > 50% high. The random-effects model was used in this analysis. The random effects model has wider confidence intervals and provides more conservative and robust results than the fixed-effect model, better accounting for inter-study differences. Data analysis was performed on an intention-to-treat basis. Sensitivity analyses were performed to assess the contribution of each study to the pooled estimation by excluding individual trials one at a time and recalculating the pooled RR estimation for the remaining studies. Publication bias was assessed using the Egger linear regression test and visual inspection of funnel plots. All analyses were performed using Review Manger Software (Review Manager (RevMan) [Computer program]. Version 5.3. Copenhagen: The Nordic Cochrane Centre, The Cochrane Collaboration, 2014).

### Trial Sequential Analysis (TSA)

Cumulative meta-analyses are prone to produce type I and type II errors because of repeated testing of significance as trial data accumulates. Statistically significant small trials are often overruled when results from adequately powered and bias-protected trials emerge^19,20^. TSA is similar to interim analyses in a single trial in which monitoring boundaries are used to determine whether a trial could be terminated early when a *P* value is sufficiently small to show the anticipated effect. Analysis was performed using Trial Sequential Analysis Viewer (0.9.5.9 Beta) anticipating a 25% relative risk reduction for efficacy outcome, α = 5% and 1 − β = 80% and estimating the required diversity adjusted information size. This methodology has been described in detail^21,22^.

## Outcomes

### Search results and bias assessment

**Figure 1:**
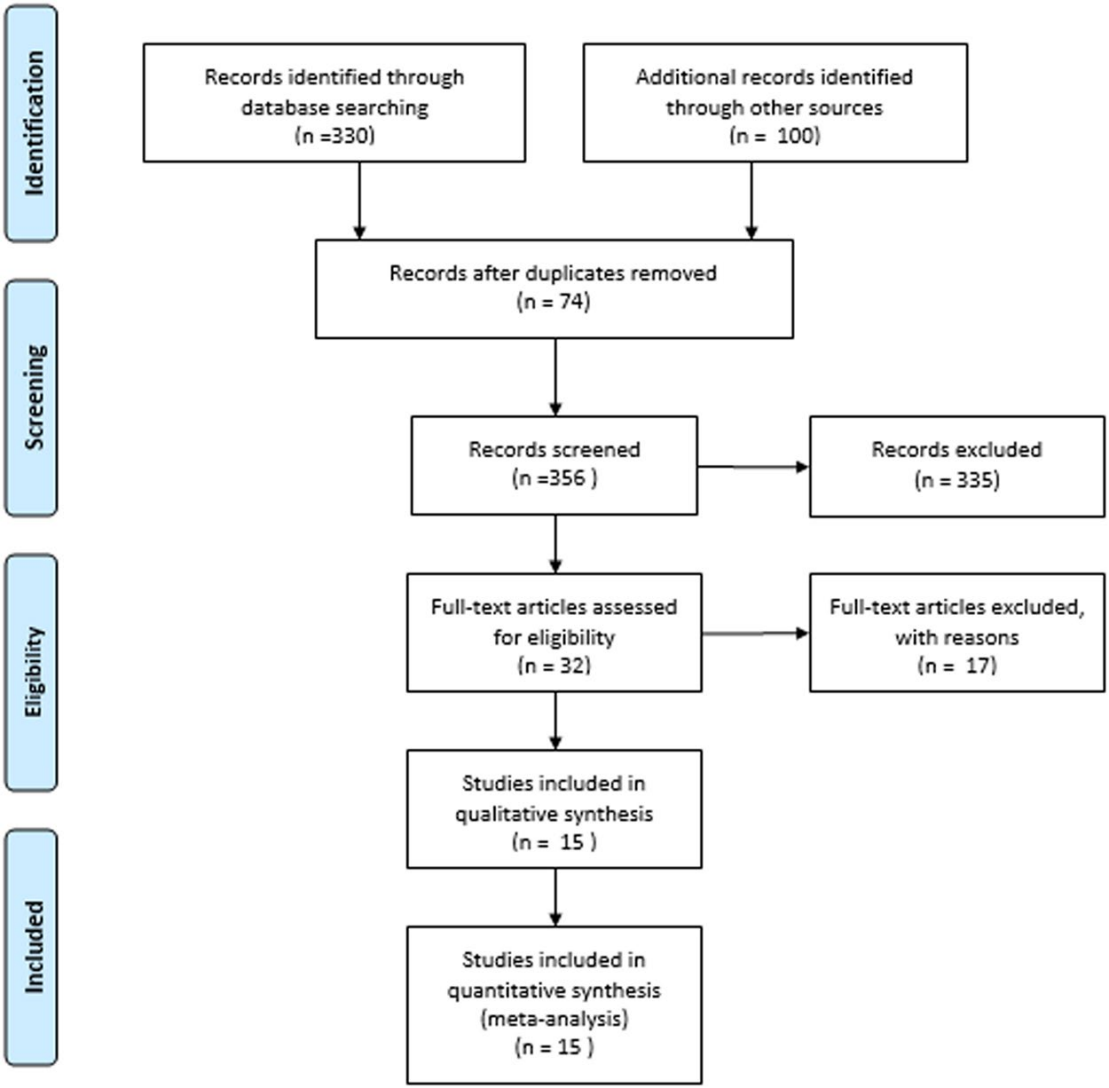
Flow diagram of literature searched for meta-analysis.

As reported in Fig. 1, the combined search strategy identified 273 potential relevant manuscripts, 22 studies were finally retrieved for more detailed assessment. Finally, 15 RCTs were included in this meta-analysis, covering a total of 2606 patients^11–13,15–17,23–31^. Characteristics of the included trials were shown in Table 1. Clinical heterogeneity was mostly due to differences in inclusion criteria, left ventricular ejection fraction (LVEF), levosimendan dose, traditional treatment, and duration of follow-up. Here, 10 studies used levosimendan in CABG. The 5 remaining studies included CABG and valve surgery. LVEF varied between 18% and 50%. Dose varied between 10 and 12 μg/kg as intravenous bolus and between 0.025 and 0.2 μg/kg as a continuous infusion. The duration of follow-up varied greatly as well. Most studies were confined to hospitalization. Six trials were multi-center. We used the Cochrane Reviewer’s Handbook 4.2 to assess risk of bias (Supplementary Fig. 1). Study quality appraisal indicated that studies were of variable quality and that 5 of them had a low risk of bias.

**Table 1:** Characteristics of included studies.

Study	Design	Year (L vs. C)	LVEF (L vs. C)	Characteristics of included patients	Time of administration	Bolus Dose	Continuous infusion dose	Control group	Follow-up
Al-Shawaf ^23^	Single-center	61/58	29/31	Type 2 diabetic Patient, CABG, LOCS, LEVF ≤ 35%	after surgery	12 μg/kg bolus over 10 minutes	0.1–0.2 μg/kg/min over 24 hours	50 ug/kg bolus over 10 minutes, followed by 0.3–0.5 μg/kg/min over 24 hours	In-hospital
Anastasiadis^24^	Single-center	61/62	36/38	CABG, LVEF ≤ 40%	before surgery	None	0.1 μg/kg/min for 24 h	Placebo	In-hospital
Cholley^15^	Multi-center	69/67	—	CABG, LVEF ≤ 40%	after anesthetic induction	None	0.1 μg/kg/min for 24 h	Placebo	180 d
Kandasamy^27^	Single-center	55/55	—	CABG, moderate to severe LV dysfunction	after anesthetic induction	None	levosimendan at 0.1 μg/kg/min for 24 h	dobutamine 5 μg/kg/min for 24 h	In-hospital
Landoni^16^	Multi-center	66/66	50/50	cardiac surgery with LVEF <25%, IABP or high-dose inotropic support	after anesthetic induction	None	0.025 to 0.2 μg/kg/min for 24 h	Placebo	180 d
Mehta^17^	Multi-center	65/65	26/27	Cardiac surgery, LEVF ≤ 35%	after anesthetic induction	0.2 μg/kg/min for 1 h	0.1ug/kg/min for 24 h	Placebo	90 d
Baysa^25^	Single-center	57/58	35/38	mitral valve surgery with LVEF ≤ 45%	after surgery	6 μg/kg bolus over 10 minutes	0.1ug/kg/min for 24 hours	standard inotropic agents	30 d
Stefan 2007	Single-center	67/69	24/27	Cardiac surgery, LEVF ≤ 30%	during surgery	None	0.1 μg/kg/min for 24 h	milrinone 0.5 mg/kg/min	In-hospital
Erb^12^	Single-center	70/63	22/22	CABG, LVEF ≤ 30%		None	0.1 μg/kg/min for 24 h	water-soluble vitamin	180 d
Eriksson^26^	Multi-center	64/64	36/36	CABG, LVEF ≤ 50%	after anesthetic induction	12 μg/kg bolus over 10 minutes,	0.2 μg/kg/min for 24 hours	Placebo	30 d
Leppikangas^28^	Single-center	75/76	69/63	high-risk cardiac surgery, LVEF ≤ 50% or LV hypertrophy	before surgery	12 μg/kg bolus over 10 minutes,	0.1–0.2 μg/kg/min for 24 hours	Placebo	In-hospital
Levin^13^	Multi-center	64/63	18/19	CABG, LVEF ≤ 25%	before surgery	Loading dose 10 μg/kg for 1 h	0.1 μg/kg/min for 23 h	Placebo	In-hospital
Shah^31^	Single-center	60/61	22/23	CABG, LVEF <30%	before surgery	None	200 μg/kg for 24 h	Placebo	In-hospital
Lomivorotov^30^	Single-center	58/57	30/30	CABG, LVEF ≤ 35%	after anesthetic induction	12 μg/kg over 10 minutes,	0.1 μg/kg/min for 24 h	IABP	In-hospital
Levin^29^	Multi-center	62/62	37/38	CABG, LOCS	after surgery	10 μg/kg for 1 h,	0.1 μg/kg/min for 24 h,	dobutamine 5–12.5 μg/kg/min	In-hospital

L: levosimendan group; C; control group; LVEF; left ventricular ejection fraction; CPB: cardiopulmonary bypass; CABG: coronary artery bypass grafting; LOCS: low cardiac output syndrome.

## Quantitative Data Synthesis

### Perioperative mortality

**Figure 2:**
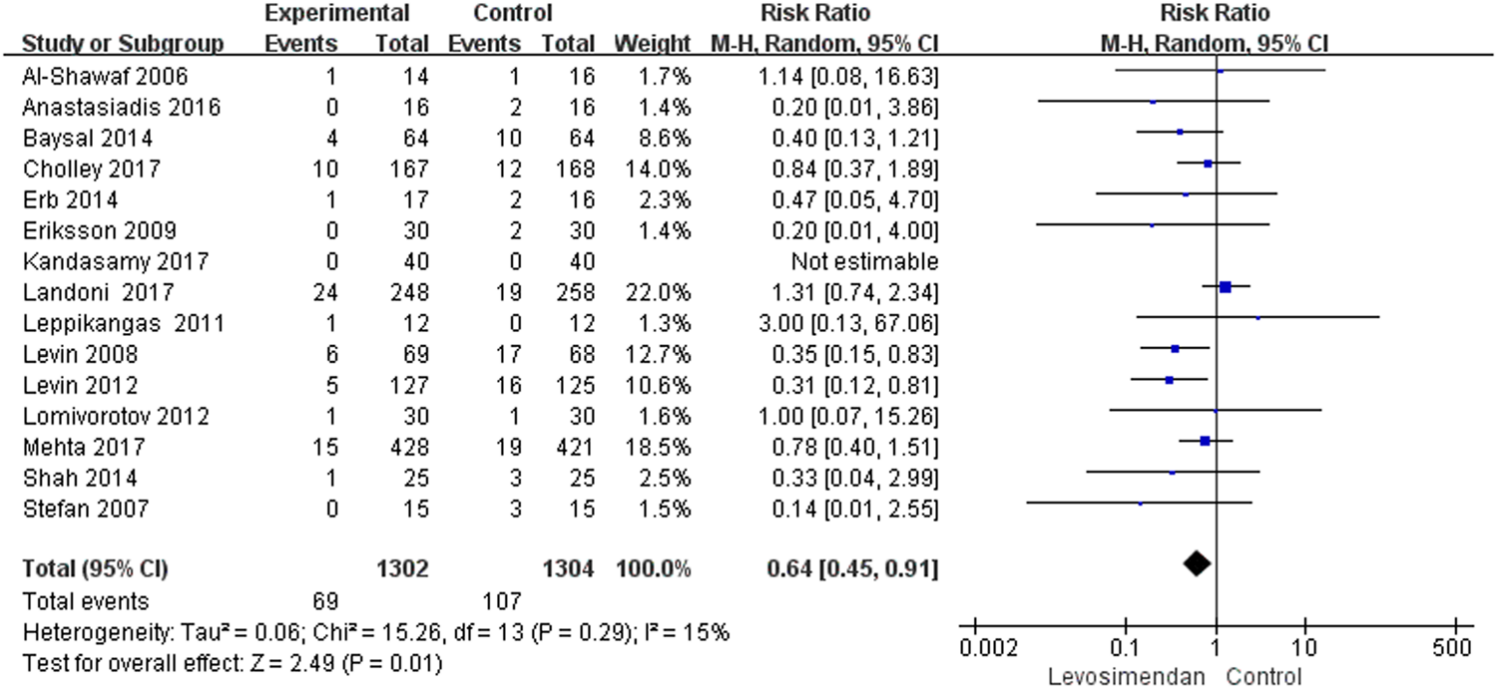
Levosimendan treatment vs. conventional treatment for the outcome of perioperative mortality.

**Figure 3:**
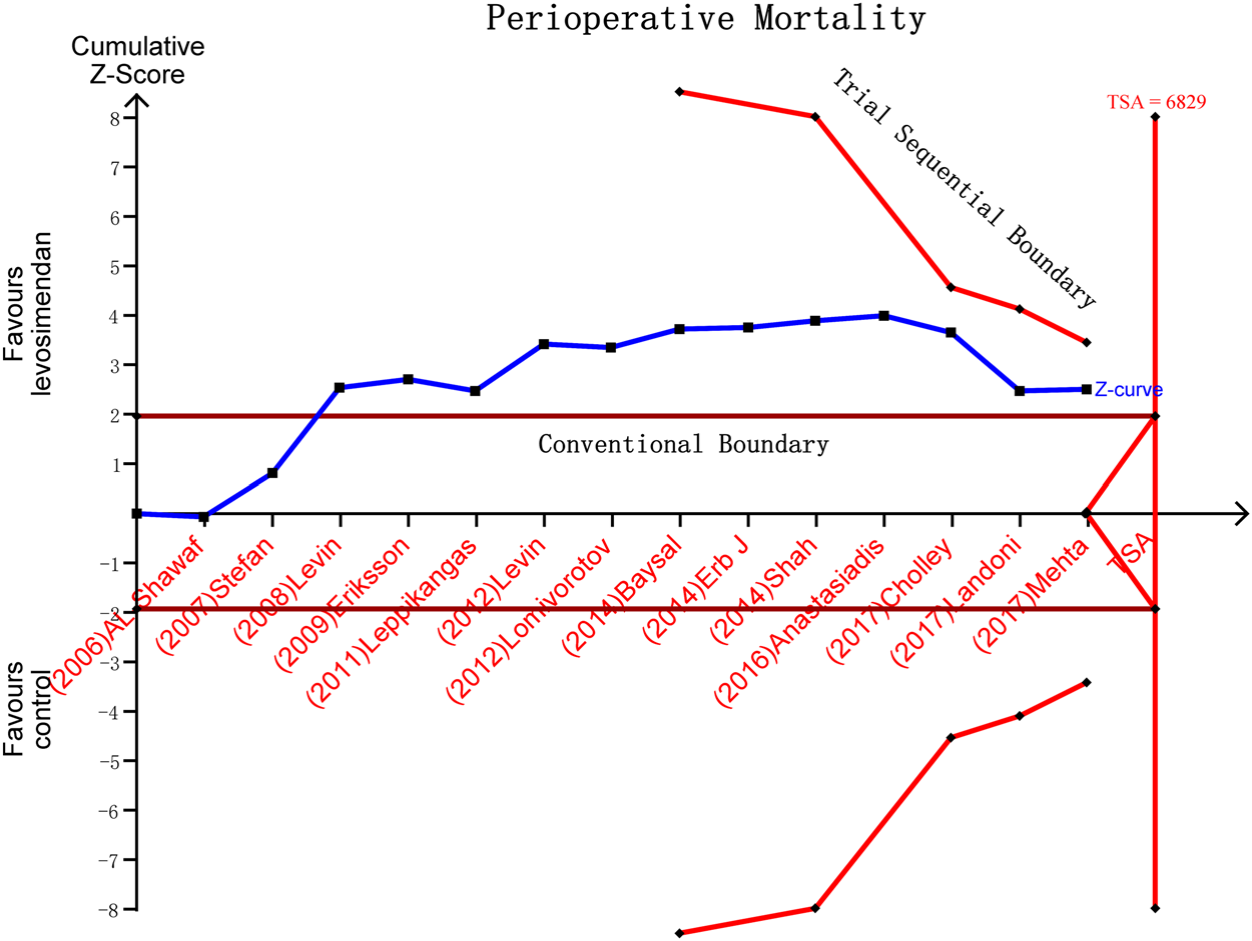
Trial sequential analysis (TSA) for the outcome of perioperative mortality.

Our analysis showed that use of levosimendan in patients with LVD undergoing cardiac surgery was associated with a significant reduction in perioperative mortality (RR: 0.64, 95%CI:0.45–0.91, *P* = 0.01, I^2^ = 15%) (Fig. 2). However, in the TSA, the cumulative Z-curve crossed the traditional boundary (*P* = 0.05) but not the trial sequential monitoring boundary, indicating lack of a firm evidence for a 25% reduction in perioperative mortality with levosimendan treatment compared with traditional treatment (Fig. 3). The small sample size was not large enough to draw this conclusion that levosimendan reduced perioperative mortality. Sensitivity analyses were performed by excluding individual trials one at a time and recalculating the pooled RR estimation for the remaining studies, which indicated that both Levin^29^ and Levin^13^ could influence the overall effect (Table 2). In this way, the results of sensitivity analysis and TSA showed that this result is not solid. Subgroup analysis was performed to produce more robust results (Table 3). The results of subgroup differed greatly. The reduction in mortality was confirmed when the studies comparing levosimendan with other inotropic agents (catecholamines and phosphodiesterase type 3 [PDE-3] inhibitors) were included (RR:0.37, 95%CI:0.19–0.69, *P* = 0.003, I^2^ = 0%). However, compared with placebo, levosimendan did not reduce perioperative mortality (RR:0.75, 95%CI:0.49–1.14, *P* = 0.17, I^2^ = 18%). Multi-center studies did not demonstrate that levosimendan could reduce perioperative mortality (RR:0.75, 95%CI:0.39–1.09, *P* = 0.10, I^2^ = 53%). Studies with levosimendan loading bolus showed that levosimendan could improve clinical outcomes (RR:0.51, 95%CI:0.34–0.77, *P* = 0.001, I^2^ = 0%). The reduction in mortality was not confirmed in patients undergoing valve surgery (RR:0.64, 95%CI: 0.12–3.38, *P* = 0.6, I^2^ = 31%). However, perioperative mortality was lower in patients undergoing CABG (RR:0.45, 95%CI: 0.29–0.71, *P* = 0.0005, I^2^ = 0%).

**Table 2:** Sensitivity analysis of perioperative mortality.

Excluded study	RR	95%CI	I^2^	Benefit *(P-*value)
Al-Shawaf ^23^	0.62	0.42–0.90	21%	0.01
Stefan 2007	0.65	0.46–0.93	15%	0.02
Levin^29^	0.73	0.52–1.02	5%	0.07*
Eriksson^26^	0.64	0.45–0.93	18%	0.02
Leppikangas^28^	0.62	0.43–0.89	17%	0.010
Lomivorotov^30^	0.62	0.42–0.91	21%	0.01
Levin^13^	0.73	0.53–1.01	3%	0.06*
Baysal^25^	0.66	0.45–0.97	16%	0.03
Shah^31^	0.64	0.45–0.93	19%	0.02
Erb^12^	0.63	0.43–0.92	21%	0.02
Anastasiadis^24^	0.64	0.45–0.93	18%	0.02
Mehta^17^	0.59	0.38–0.90	20%	0.02
Landoni^16^	0.54	0.38–0.76	0	0.000
Cholley^15^	0.59	0.39–0.89	20%	0.01
Kandasamy^27^	0.64	0.45–0.91	15%	0.01

*When we excluded Levin^29^ or Levin^13^, the results had no statistically significant differences.

**Table 3:** Subgroup analysis of perioperative mortality.

Subgroup of interest	Event Levosimendan	Event Traditional Treatment Group	Risk Ratio (95%CI)	*P*	I^2^
Studies with levosimendan bolus	33 of 774	66 of 766	0.51 (0.34–0.77)	0.0007	0
Studies without levosimendan bolus	36 of 528	41 of 538	0.91 (0.56–1.49)	0.63	6%
Multi-center studies	60 of 1069	85 of 1070	0.75 (0.39–1.09)	0.10	53%
Single-center studies	9 of 223	22 of 234	0.46 (0.22–0.97)	0.04	0
Patients undergoing CABG	25 of 535	56 of 534	0.45 (0.29–0.71)	0.0005	0
Trials comparing levosimendan with placebo	57 of 1070	75 of 1071	0.75 (0.49–1.14)	0.17	18%
Trials comparing levosimendan with other inotropic agents	11 of 202	31 of 203	0.37 (0.19–0.69)	0.002	0%
Patients undergoing valve surgery	5 of 76	10 of 76	0.64 (0.12–3.38)	0.60	31%

### Secondary endpoints

Renal-replacement therapy was lower in the levosimendan group in random effects (RR:0.71, 95%CI:0.52–0.95, *P* = 0.01, I^2^ = 0%) (Supplementary Fig. 2). However, in the TSA, the cumulative Z-curve crossed the traditional boundary (*P* = 0.05) but not the trial sequential monitoring boundary, indicating lack of a firm evidence for a 25% reduction in renal replacement with levosimendan treatment compared with traditional treatment (Supplementary Fig. 3). The small sample size was not large enough to draw this conclusion that levosimendan reduced the incidence of renal-replacement treatment. We performed sensitivity analyses and found Levin^29^, Levin^13^, Baysal^25^, and Mehta^17^ all affected the overall effect (Supplementary Table 1). All of this evidence indicated that there was not enough evidence to support the idea that levosimendan could reduce renal-replacement therapy. Levosimendan did not reduce the incidence of atrial fibrillation (RR:0.82 95%CI: 0.64–1.07, *P* = 0.38, I^2^ = 66%)(Supplementary Fig. 4), myocardial infarction (RR:0.56, 95%CI:0.26–1.23, *P* = 0.15, I^2^ = 33%) (Supplementary Fig. 5), or ventricular arrhythmia (RR:0.74, 95%CI:0.49–1.11, *P* = 0.14, I^2^ = 45%) (Supplementary Fig. 6). Levosimendan increased the incidence of hypotension (RR:1.11,95%CI:1.00–1.23, *P* = 0.14, I^2^ = 0%) (Supplementary Fig. 7).

## Discussion

This meta-analysis, which contained the largest number of patients with LVD undergoing cardiac surgery of any such analysis, demonstrated that there is no solid evidence suggesting that levosimendan treatment could reduce perioperative mortality, renal replacement treatment and atrial fibrillation, myocardial infarction, or ventricular arrhythmia. In fact, levosimendan might increase the incidence of hypotension.

Previous trials have demonstrated that levosimendan can increase cardiac stroke volume without increasing myocardial oxygen demand, and reduce peripheral resistance^32^, and levosimendan treatment was associated with lower incidence of perioperative LCOS and atrial fibrillation, shorter mechanical ventilation and ICU stays, and lower 30-day mortality relative to traditional treatments among LVD patients undergoing cardiac surgery^29,33^. However, most trials were small, single-center studies without robust evidence. The 3 recent large randomized clinical trials (LEVO-CTS, CHEETAH, and LICORN) provided new evidence. None of these 3 trials indicated that levosimendan had benefits with respect to clinical outcomes, which was consistent with our analysis^15–17^.

There are many possible reasons for the heterogeneous results of studies with levosimendan in the situation of cardiac surgery. Other inotropic agents (catecholamines and phosphodiesterase type 3 [PDE-3] inhibitors) are associated with an increase in morbidity and mortality. The benefits of levosimendan may be attributed to decreased usage of other inotropic agents in previous studies. As suggested in previous clinical trials and meta-analyses, levosimendan may benefit only patients who had severe LVD at baseline, and needed more inotropic agents^17,34^. Many confounding factors, such as patient’s baseline characteristics, coexisting diseases, medications, or surgeon’s experience may affect the outcomes. With the advancement of surgical techniques, the declining death rate makes it harder to demonstrate a minor benefit of additional therapy with regard to overall mortality. An adequately convincing clinical study evaluating the effect of levosimendan treatment on perioperative mortality would require more patients, including high-risk patients. All of these factors contributed to the varied outcomes.Our meta-analysis did not find that levosimendan infusion could reduce the incidence of atrial fibrillation in patients with LVD undergoing cardiac surgery. The effects of levosimendan on atrial fibrillation differed greatly. The recent large randomized clinical trials (LEVO-CTS, CHEETAH, and LICORN) did not indicate an increased incidence of atrial fibrillation in the levosimendan group. However, the REVIVE II study reported a greater rate of atrial fibrillation in the levosimendan group^35^. Given this confusing situation, more clinical trials are needed.Although our meta-analysis did not demonstrate that levosimendan treatment was associated with a significant reduction in renal replacement treatment relative to traditional treatment, levosimendan groups tended to have fewer patients undergoing renal replacement therapy, even when Levin^29^ and Levin^13^ were excluded. Fedele *et al*. demonstrated that levosimendan has both an immediate renoprotective effect, mediated by a selective arterial and venous renal vasodilating action, and a slower cardiac-enhancing function^32^. Yakut N *et al*. also found that levosimendan significantly alleviated ischemia reperfusion injury in the renal tubules^36^. However, all of these studies had very small sample sizes, which does not lend confidence to their conclusions. Large clinical trials with convincing evidence are needed to resolve this confusing situation.

## Limitations

Our conclusions should be viewed in the context of the limitations of this work. First, although there was no apparent heterogeneity in statistical analysis, the heterogeneity in clinical trials and methodology were inevitable. This included different risk profiles of the included patients, and varied dosage of levosimendan. Second, although we performed an extensive search strategy, some trials might not be included in our meta-analysis. However, this meta-analysis is the largest population-based analysis of levosimendan in patients with LVD undergoing cardiac surgery. More clinical trials are needed to evaluate the effects of levosimendan.

## Conclusion

There was not enough high-quality evidence to either support or contraindicate the use of levosimendan in cardiac surgery patients with LVD.

## Electronic supplementary material

supplementary file
