# Supplementary material for: Levosimendan in patients with left ventricular dysfunction undergoing cardiac surgery: a meta-analysis and trial sequential analysis of randomized trials
Source: Sci Rep. 2018 May 17;8:7775. doi: 10.1038/s41598-018-26206-w (PMC5958056; doi:10.1038/s41598-018-26206-w)

# Levosimendan in patients with left ventricular dysfunction undergoing cardiac surgery: a meta-analysis and trial sequential analysis of randomized trials

Zhenhua Xing, MD^1^, Liang Tang, MD^1^, Pengfei Chen, MD^1^, Jiabing Huang, MD^1,^, Xiaofan peng,MD^1^, Xinqun Hu, MD^1^*

**Supplementary Table 1: Sensitivity analysis of renal replacement treatment**

| Excluded study | RR | 95%CI | I^2^ | benefit (p-value) |
| --- | --- | --- | --- | --- |
| Al-Shawaf 2006 | 0.70 | 0.5-0.97 | 8% | 0.03 |
| Levin 2008 | 0.74 | 0.54-1.00 | 0% | 0.05* |
| Levin 2012 | 0.73 | 0.54-1.00 | 0% | 0.05* |
| Baysal 2014 | 0.71 | 0.50-1.00 | 16% | 0.05* |
| Shah 2014 | 0.68 | 0.49-0.95 | 19% | 0.02 |
| Erb 2014 | 0.70 | 0.50.-0.99 | 8% | 0.04 |
| Anastasiadis 2016 | 0.64 | 0.45-0.93 | 18% | 0.02 |
| Mehta 2017 | 0.73 | 0.51-1.03 | 20% | 0.07* |
| Landoni 2017 | 0.66 | 0.44-1.00 | 8 | 0.05 |
| Cholley 2017 | 0.61 | 0.44-0.85 | 0 | 0.85 |
| Lomivorotov 2012 | 0.72 | 0.53-0.97 | 0% | 0..03 |
| Kandasamy 017 | 0.64 | 0.45-0.91 | 15% | 0.01 |

* when we excluded Levin 2008, Levin 2012, Baysal 2014, or Mehta 2017, the effect has no statistical significance

**Supplementary Fig1. Bias assessment using Cochrane Reviewer’s Handbook 4.2**


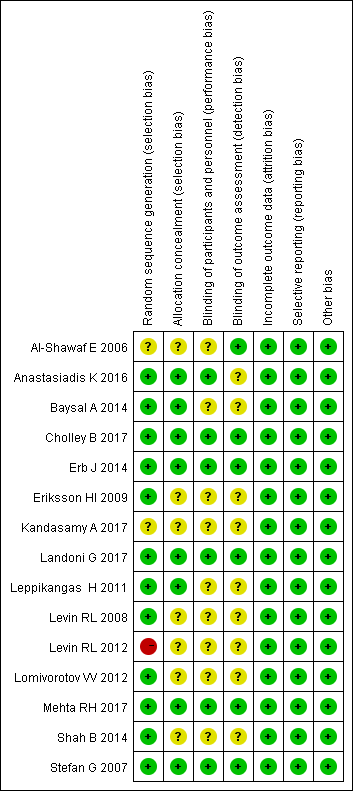


**Supplementary Fig2.** **levosimendan group vs conventional treatment for the outcome of renal replacement treatment**


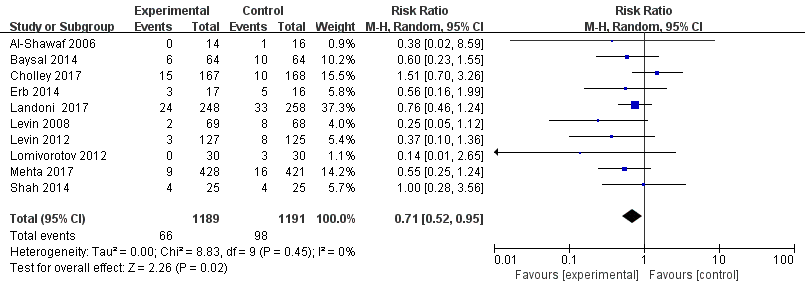


**Supplementary Fig 3. Trial sequential analysis (TSA) for the outcome of renal replacement treatment.**


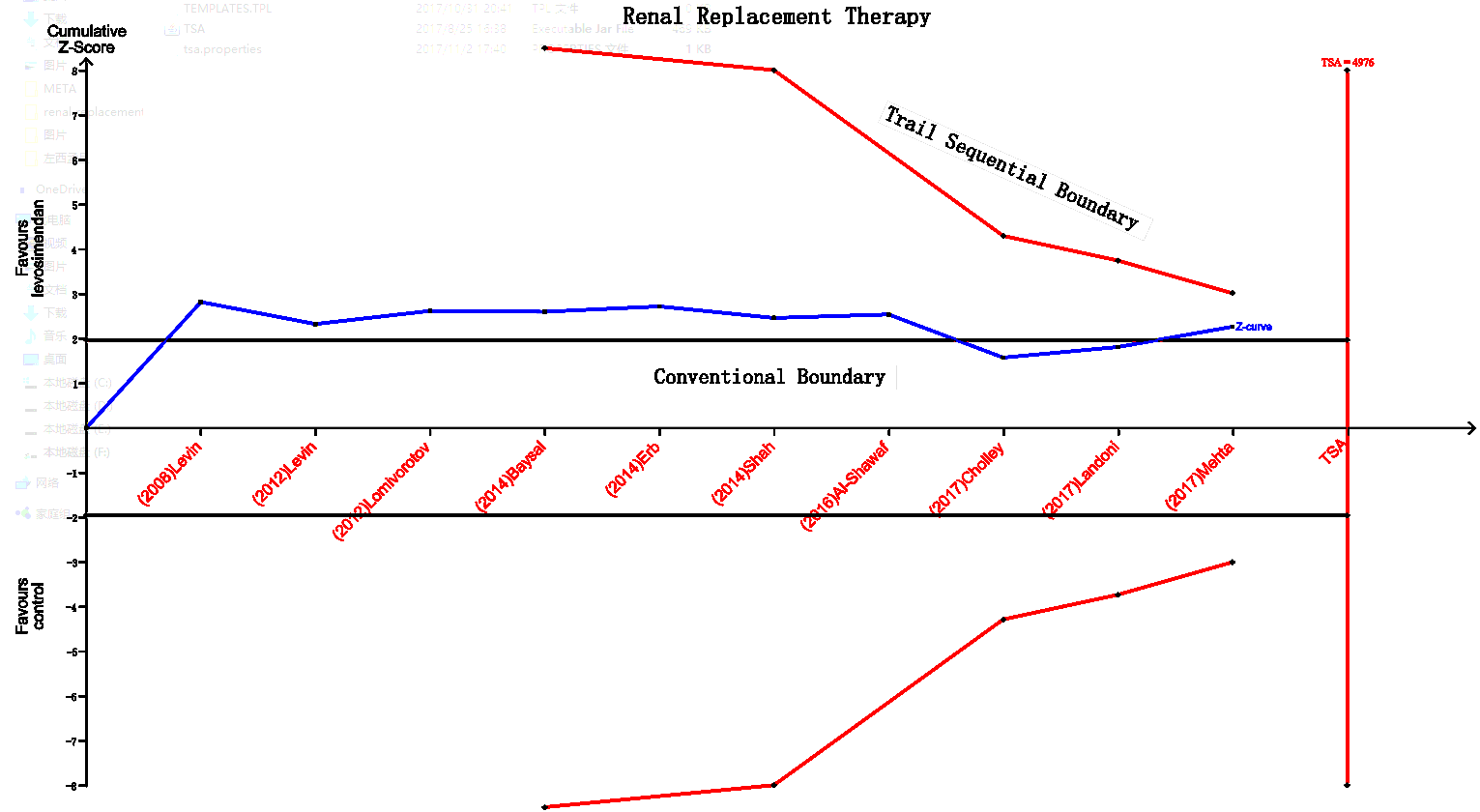


**Supplementary Fig 4. levosimendan group vs conventional treatment for the outcome of atrial fibrillation**


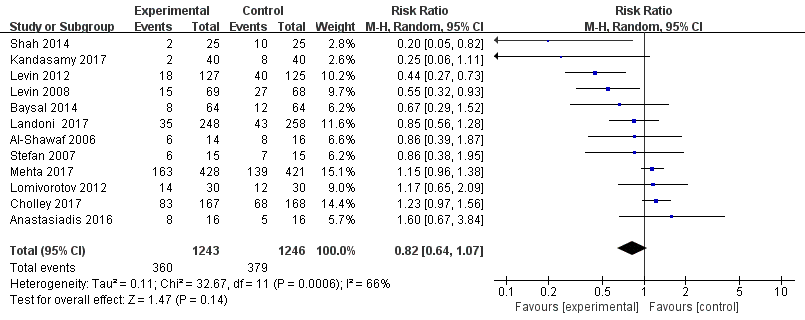


**Supplementary Fig 5. levosimendan group vs conventional treatment for the outcome of myocardial infarction**


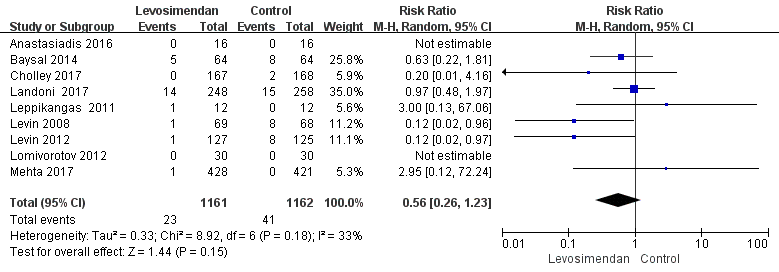


**Supplementary Fig 6. levosimendan group vs conventional treatment for the outcome of ventricular arrhythmia**


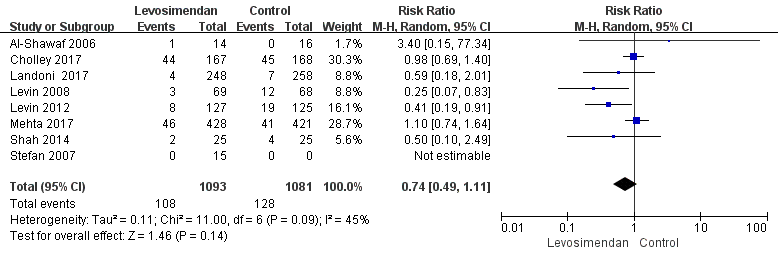


**Supplementary Fig 7. levosimendan group vs conventional treatment for the outcome of ventricular arrhythmia**


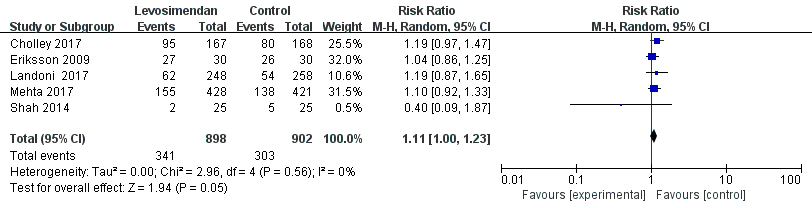

Supplement: Supplementary file 1 — supplementary file [file 41598_2018_26206_MOESM1_ESM.docx]
